# Green low-cost synthesis of zero-valent iron nanoparticles from *Palm Petiole Extract* for Cr(VI) removal from water

**DOI:** 10.1007/s11356-024-34092-1

**Published:** 2024-06-28

**Authors:** Dhiss Tesnim, Ben Amor Hédi, Djellabi Ridha, Antonio Cid-Samamed

**Affiliations:** 1 National School of Engineers of Gabes, Laboratory of Research: Processes, Energy, Environment & Electrical Systems PEESE (LR18ES34), University of Gabes, Gabes, Tunisia; 2 Department of Chemical Engineering, Universitat Rovira i Virgili, 43007 Tarragona, Spain; 3 Faculty of Sciences, Physical Chemistry Department, University of Vigo, 32004 Ourense, Spain

**Keywords:** Biomass valorization, Cr(VI) adsorption, Green synthesis, P-NZVI nanoparticles, Water remediation

## Abstract

One of the hottest research topics over the last decades was the valorization or/and recycling of agro-industrial wastes into different valuable liquid or solid products, which is considered a sustainable and low-cost approach. In this study, we developed zero-valent iron nanoparticles from *Palm Petiole Extract* (P-NZVI) using a green and straightforward approach. The as-synthesized P-NZVI was used to adsorb Cr(VI) in water. The physico-chemical characterizations of P-NZVI, including the particle size, crystalline structure, surface area, morphology, and functional groups, were investigated via several techniques such as UV-vis spectroscopy, SEM, TEM, XRD, FTIR, AFM, DLS, pH_ZPC_ measurement, and BET analysis. The adsorption performance of P-NZVI was studied under different operational parameters, including pollutant concentration, pH, temperature, and adsorbent mass. The adsorption rate was found to be 89.3% within 40 min, corresponding to the adsorption capacity of 44.47 mg/g under the following conditions: initial Cr(VI) concentration of 40 mg/L, pH 5, and a P-NZVI dosage of 1 g/L. It was found that the adsorption pattern follows the Langmuir and the pseudo-second-order kinetic models, indicating a combination of monolayer adsorption and chemisorption mechanisms. The thermodynamic study shows that the adsorption process is endothermic and spontaneous. The reusability of P-NZVI was carried out four times, showing a slight decrease from 89.3 to 87%. These findings highlight that P-NZVI’s could be an effective green adsorbent for removing Cr(VI) or other types of toxic pollutants from water.

## Introduction

Due to the high toxicity, mobility, and wide mobility of hexavalent chromium (Cr(VI)) in wastewater, a lot of research studies have been reported for developing processes or/and materials to remove it from water (Jaishankar et al. 2014; Elahi et al. 2020). The discharge of Cr(VI)-polluted wastewater is carried out from various industrial sectors such as electroplating and mining, dye and textile, anti-corrosion materials, and ceramic glazers (Velusamy et al. 2022). Its persistence in aquatic environments hampers practical mitigation efforts, impacting ecosystems and aquatic life (Mohanty et al. 2023). Moreover, Cr(VI) is designated as a group 1 carcinogen by the World Health Organization, underlining its severe health risks for humans (Chen et al. 2022). The ingestion of water containing excessive levels of Cr(VI) can lead to detrimental health effects, with fatal consequences observed at concentrations surpassing 0.1 mg/g of body weight (Mortada et al. 2023). As a result, stringent regulations, such as the Drinking Water Directive, have been established to limit chromium levels and protect both environmental integrity and public health (Organization 2020).

To reduce the amount of Cr(VI) released into the environment and lessen its detrimental effects on ecosystem health and human well-being, strict environmental regulations must be implemented immediately. Recently, research has focused on sustainable and environmentally friendly methods to remove pollutants from wastewater to protect human health and natural resources for the next generations (Bhavya et al. 2021; Saravanan et al. 2022). Various techniques have been used to remove Cr(VI) from aqueous solutions, such as adsorption (KS et al. 2023), chemical precipitation (Qasem et al. 2021), ion exchange (Bashir et al. 2019), electrocoagulation (Ayub et al. 2020), and electrodialysis (Wan et al. 2019). According to a literature review, the adsorption technique is a workable, low-cost, simple, and promising method for removing Cr(VI) from aqueous solutions (Rajapaksha et al. 2022). Several adsorbing materials have been used, such as activated carbon (Liang et al. 2019), zero valent-iron nanoparticles (NZVI) (Rashtbari et al. 2021), clay (Bansal and Purwar 2021), biochar (Li et al. 2022c), polymer (Valizadeh et al. 2020), metal-organic frameworks (Yousefi et al. 2024), and other cost-effective adsorbents (Wang et al. 2022; Wen et al. 2022).

Recently, NZVI has received much attention, primarily due to its effective removal of contaminants from aqueous solutions at low cost. However, traditional methods for synthesizing NZVI often require the use of huge amounts of solvents, reagents, and intensive energy, making the synthesis process less or unsustainable.

These chemicals may include sodium borohydride (NaBH_4_) (Al-Graiti et al. 2022), ethylene glycol (Ruiz-Torres et al. 2019), and carbothermal synthesis (Nisticò and Carlos 2019). Recently, some research groups developed sustainable NZVI synthesis processes as alternatives to chemical-based approaches (Latif et al. 2019; Singh et al. 2020). According to Wang et al., NZVI is a green material with high treatment efficiency, controlled particle size (1–100 nm), and numerous activation sites (Wang et al. 2014). Interestingly, the valorization of agro-wastes into NZVI has been reported by different studies using various types of starting agro-substrates, providing an alternative to conventional routes (Desalegn et al. 2019). Several agro-wastes have been utilized, such as *Green Tea* ((Eddy et al. 2022), *Black Tea* (Souza et al. 2020), *waste Palm Petiole* (Tesnim et al. 2023), *Eucalyptus leaf* (Wang et al. 2014), and *Ricinus Communis* (Abdelfatah et al. 2021). Numerous studies highlight the role of compounds such as polyphenols, proteins, alkaloids, phenolic acids, sugars, and terpenoids as biological reducing and stabilizing agents in the synthesis of NZVI (Ingle 2021; Poudel et al. 2022). Notably, natural antioxidants from plants, particularly polyphenols, have garnered attention for their affinity for proteins and metal ions and their reducing properties (Jaiswal et al. 2012). Among the hydroxybenzoic acid derivatives, gallic acid is recognized as an organic phenolic compound with potential applications (Bodoira and Maestri 2020).

The peculiar structural and electrical characteristics of synthesized NZVI make it a potential material for the removal of toxic pollutants from wastewater, such as antibiotics (Hamad and El-Sesy 2023), pesticides (Dhir 2021), and dye pollutants (Eddy et al. 2022). Additionally, NZVI-based materials proved to be excellent adsorbents for the removal of heavy metals (Fu et al. 2015). Due to their high reactivity, significant surface area, and potent reduction or immobilization of heavy metal ions, NZVIs have attracted much interest in Cr(VI) treatment (Alazaiza et al. 2022; Di et al. 2023). Several studies have elucidated the potential effects of NZVI; these investigations have delved into the toxicity of NZVI on aquatic organisms, revealing that elevated concentrations of NZVI led to detrimental impacts on plants, fish, algae, and invertebrates. These adverse effects included decreased survival rates, inhibited growth, and alterations in the behavior of the organisms under examination (Li et al. 2016; Sun et al. 2022; Mohanty et al. 2023).

Herein, we report the green synthesis of P-NZVI using *Palm Petiole Extract* as a green reducing agent to remove Cr(VI) from water. The process used in this study is characterized by its simplicity and efficiency, contrasting with traditional methods like hot extraction (Zayed et al. 2023, p. 60), cold extraction (Pattanayak et al. 2021), microwave irradiation (Kangralkar and Jayappa 2020), and Soxhlet apparatus (Wu et al. 2019) commonly employed for P-NZVI synthesis. The key innovation lies in extracting *Palm Petiole* using a coffee maker, offering a sustainable alternative to conventional methodologies. Batch experiments were conducted to explore the influence of key parameters: pH, initial Cr(VI) concentration, P-NZVI dosage, and temperature on Cr(VI) removal. Various analytical techniques, including pH_ZPC_, DLS, AFM, SEM, BET, TEM, XRD, and FTIR, were employed to characterize P-NZVI. The main objective of this investigation is to develop a green approach to the synthesis of P-NZVI and study the effect of operating factors on removing Cr(VI) from water.

## Materials and methods

### Materials

Ferric chloride hexahydrate (FeCl_3_, 6H_2_O) was procured from PROLABO. The 1,5-diphenyl carbohydrazide and the potassium dichromate (K_2_Cr_2_O_7_) were purchased from Sigma-Aldrich Company. Thermo Fisher Scientific supplied Sulphuric acid (H_2_SO_4_) and sodium hydroxide (NaOH).

### Methods

#### Collection of waste palm

Palm waste was collected from a local farm in Gabes (southern Tunisia). The material was carefully washed, crushed, and then stored at 20 °C. The crushing and drying of palm waste significantly reduced its moisture content. The utilized palm waste was preserved in amber bottles at a temperature of 4 °C before being considered for use.

#### Extraction of polyphenols from waste palm extract

The total phenolic content of the samples was assessed through the Prussian blue assay, following the methodology outlined in prior reports with some modifications (Margraf et al. 2015). This method was selected for its simplicity and use of readily available materials and chemicals. In each case, a 50-mL conical flask containing 2 mL of the sample extract was combined with 20 mL of distilled water, followed by adding 3 mL each of 0.008 M K_3_Fe (CN)_6_ and 0.1 M FeCl_3_ in 0.1 M HCl. After 15 min, a noticeable color change from green to blue occurred, and the absorbance was measured at 700 nm, the wavelength corresponding to the highest absorbance of the colored solution. Calibration curves were generated using gallic acid standard solutions of varying concentrations (30–150 mg/L) to quantify the total phenolic content in each sample (palm spathe, data seeds, grapes palm, trunk, leaflets, and palm petiole) via the Prussian blue assay. The total phenolic content was expressed as gallic acid equivalents (mg/g of dry extract) using the equation *y* = 0.0032*x* + 0.0214, where *y* represents the absorbance. A linear regression coefficient (*R*^2^) of 0.9872 was obtained, validating the reliability of the calibration curve.

#### Preparation of P-NZVI

**Fig. 1:**
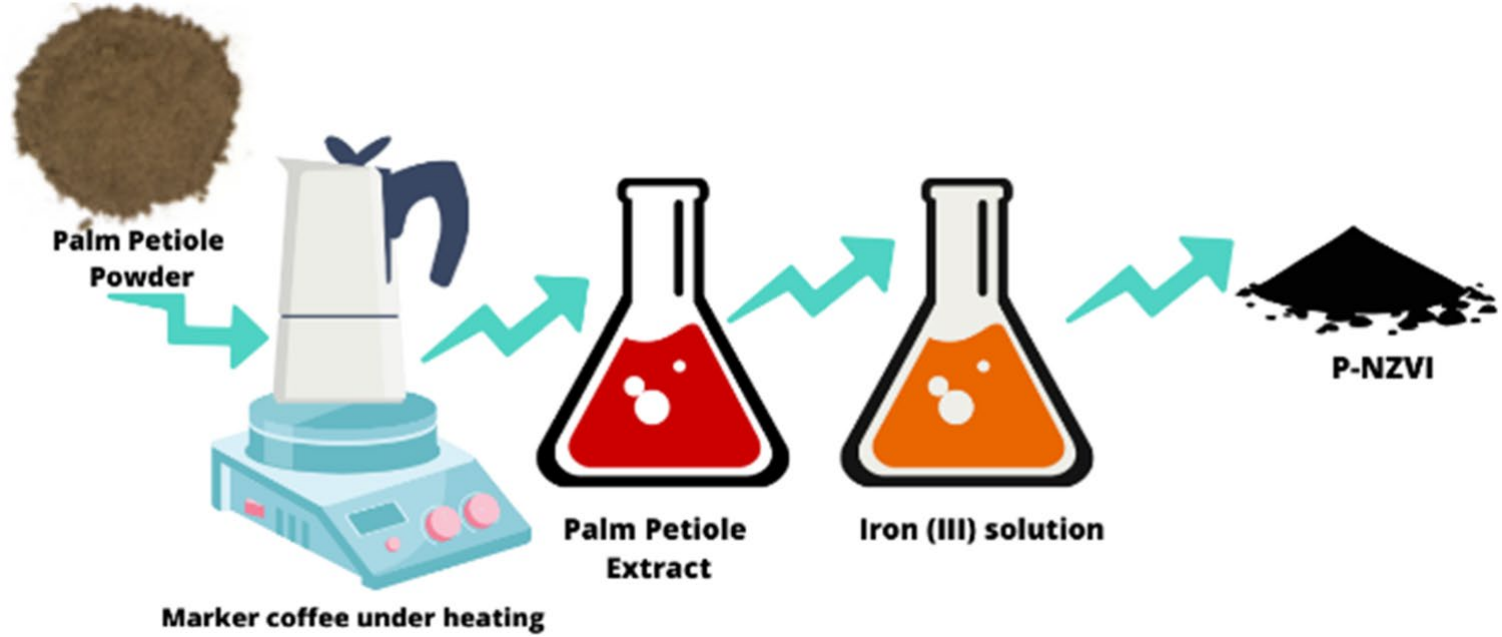
Schema shows the steps of P-NZVI synthesis

The P-NZVI nanoparticles were synthesized following a procedure used in our previous study (Tesnim et al. 2023) with some modifications. The synthesis steps of P-NZVI are depicted in Fig. 1.

### Characterization techniques

Several techniques of characterization were used to examine the nanoparticles. The absorbance spectra of the nanoparticles between 190 and 700 nm were obtained using UV-visible spectroscopy (Shimadzu UV2550, Kyoto, Japan). Dynamic light scattering (DLS) (ZEN 3600, Malvern, Worcestershire, UK), transmission electron microscopy (TEM) at 100 kV (Zeiss EM 10C), and scanning electron microscopy (SEM) (BEL SORP mini, Osaka, Japan) were utilized to investigate size and morphology. The Fourier-transform infrared spectroscopy (FTIR) spectrum (BRAIC WQF-510, Beijing, China) was interpreted to identify surface functional groups responsible for reducing, covering, and stabilizing the reduced nanoparticles. The Brunauer—Emmett—Teller (BET) analysis was performed to ascertain the materials’ specific surface area precisely. X-ray diffraction (XRD) was used to estimate and analyze a material’s crystal structure. The nanoparticles’ zero-point charge (*pH*_*ZPC*_) was determined using Rawat and colleagues’ pH drift technique (Rawat and Singh 2021) with a few adjustments.

### Adsorption of Cr(VI) removal

In this study, Cr(VI) adsorption experiments were carried out in a batch system. Several operating factors were studied, including adsorbent dosage (0.2 to 1 g/L), pH levels (2 to 10), contact time (5 to 60 min), and the initial concentration (10 to 50 mg/L). The adsorption isotherms were analyzed to obtain precise information on the efficiency of the adsorption process. Furthermore, the solution’s temperature was varied from 298 to 328 K to determine thermodynamic parameters related to the adsorption phenomenon.

#### Analytical methods

The concentration of Cr(VI) during the adsorption process was followed by calorimetrically using 1,5-diphenyl carbohydrazide method (Mortazavian et al. 2018). The concentration of Cr(VI) was measured using a UV-vis spectrophotometer (UV-1800, Shanghai, China) at a wavelength of 540 nm. Before the treatment, each sample was filtered through 0.45-μm syringe filters to remove adsorbed particles.

All studies were done in duplicate or triplicate, and the reported data are the means with a relative error of 3%.

#### Adsorption kinetics models

The study of adsorption kinetics provides valuable insights into the rate and mechanisms involved in adsorption, including mass transfer, diffusion, and surface reactions on the adsorbent material (KS et al. 2023; Qi et al. 2023). Furthermore, understanding the kinetics is critical for establishing the minimal contact time required between the adsorbent and adsorbate to attain steady-state or pseudo-equilibrium in the system (Gao et al. 2022). This knowledge has enormous practical importance since it can save time and energy. The pseudo-first-order (PFO), pseudo-second-order (PSO), and intraparticle diffusion (IPD) kinetic models are widely utilized in studying adsorption processes in the aqueous phase (Lima et al. 2021).

The PFO kinetic rate (Eq. 1) is written as

1 $${q}_t={q}_e\left(1-{e}^{-{k}_1t}\right)$$

The adsorption capacities are given as *q*_*t*_ and *q*_e_ (mg/g) for equilibrium and any contact time *t* (min), respectively. The PFO model’s rate constant is *k*_1_ (min^*−*1^).

The PSO kinetic model (Eq. 2) is as follows:

2 $${q}_t=\frac{k_2{q}_e^2t}{1+{k}_2{q}_et}$$

where *k*_2_ (g/mg min) represents the pseudo-second-order rate constant.

The IPD model (Eq. 3) is shown as follows:

3 $${q}_t={k}_{id}{t}^{\frac{1}{2}}+{C}_i$$

In the intraparticle diffusion model, *k*_*id*_ (mg/g min) represents the rate constant, and *C*_*i*_ (mg/g) is a constant related to the boundary layer thickness. A higher value of *C*_*i*_ indicates a more significant impact on the limiting boundary layer.

#### Adsorption isotherms

When the adsorbate uptake on the adsorbent surface reaches a steady state, and the remaining amount in the solution stabilizes, the adsorption process enters a state of dynamic equilibrium. To effectively design adsorption systems and better understand how solutes and adsorbents interact, the adsorption isotherm, established at a constant temperature, is essential (Li et al. 2023). To describe the Cr(VI) adsorption process onto P-NZVI in this study, two widely used adsorption isotherm models were applied (Mortazavian et al. 2018; Qi et al. 2023).

The Langmuir isotherm is founded on the assumption that adsorbed molecules, due to constant enthalpies and sorption activation energies, form a monolayer on the homogeneous surface of the adsorbent, with no interaction between them. The corresponding nonlinear (Eq. 4) is as follows:

4 $${q}_e=\frac{K_L{C}_e{q}_{max}}{1+{K}_L{C}_e}$$

where *C*_*e*_ is the adsorbate’s equilibrium concentration (mg/L), *C*_0_ is the initial concentration of the adsorbate (mg/L), and *q*_max_ (mg/g) and *K*_L_ (L/mg) are the maximum adsorption capacity and a constant associated with adsorption energy, respectively. A separation factor (*R*_L_) can also be added (Eq. 5):

5 $${R}_L=\frac{1}{1+{K}_L{C}_0}$$

The dimensionless constant *R*_L_ is a valuable parameter for determining operating conditions: *R*_L_ > 1 indicates unfavorable adsorption, *R*_L_ = 1 suggests linear adsorption, *R*_L_= 0 implies irreversible adsorption, and 0 < *R*_L_ < 1 signifies favorable adsorption.

The Freundlich isotherm is an empirical model that assumes a heterogeneous adsorption surface with molecular interactions. The following is the appropriate nonlinear (Eq. 6):

6 $${q}_e={K}_F{C}_e^n$$

In the Freundlich isotherm equation, *K*_*F*_ (mg/g)/(mg/L) represents the Freundlich constant, which characterizes the adsorption strength. The parameter *n* (dimensionless; 0 < *n* < 1) reflects the magnitude of the adsorption driving force or surface heterogeneity (the adsorption isotherm becomes linear when *n* =1, favorable when *n* < 1, and unfavorable when *n* > 1).

#### Thermodynamic studies

Thermodynamic studies help define the spontaneity of a reaction, the degree of system unpredictability, and the endothermic or exothermic nature of the reaction. The thermodynamic Eqs. 7 and 8 are as follows (Saranya et al. 2017):

7 $$\Delta {G}^{{}^{\circ}}=- RTln{k}_D$$

where ∆*G*^°^ is the Gibbs free energy, *R* is the universal gas constant (8.314 J/mol·K), *T* is the absolute temperature (K), and *k*_*D*_ is the distribution coefficient calculated by:

8 $${LnK}_D=-\frac{\Delta {H}^{{}^{\circ}}}{RT}+\frac{\Delta {S}^{{}^{\circ}}}{R}$$

With $${k}_D=\frac{q_e}{C_e}$$

where ∆*H*^°^ is the enthalpy change, ∆*S*^°^ is the entropy change, *q*_e_ is the equilibrium adsorption capacity (mg/g), and *C*_e_ is the *C*_*r*_(VI) concentration at equilibrium (mg/ L).

## Results and discussion

### Optimization of the phenolic content of waste palm

**Fig. 2:**
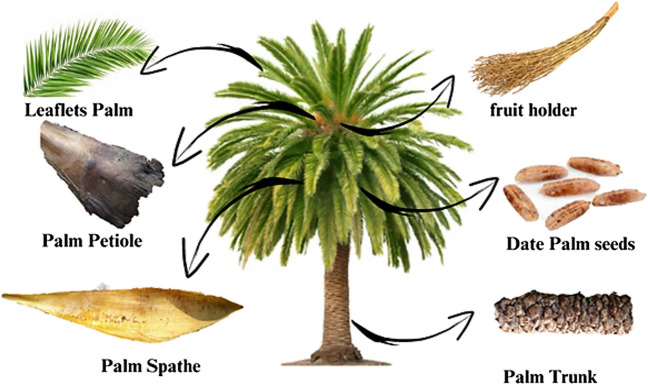
Palm tree with its different waste components

The phenolic content of different components of the date palm tree is shown in Fig. 2.

Table 1 shows that all the sample extracts exhibited positive tests for phenolic compounds. The *Palm Petiole* has the highest total polyphenol content compared to the *date seeds*, *Fruit holder*, *Leaflets*, *Palm spathe*, and *Trunk*. The raw *Palm Petiole* (PP) was used in this study.

**Table 1:** Total phenolic content of waste palm.

Waste palm	Palm spathe	Date seeds	Fruit holder	leaflets	Palm Petiole	Trunk
GAE (mg/g)	6.04	10.17	5.58	0.62	22.76	1.867

*GAE* gallic acid equivalents (mg GAE/g extract)

Based on Table 2, it can be concluded that there are variations in the phenolic content in different plants. Green tea’s polyphenol content is higher than PP’s gram content; however, due to its importance, it can be used to produce adsorbent materials. Therefore, palm petiole was selected to be valorized into valuable adsorbents. In addition, this type of waste is largely available in Tunisia.

**Table 2:** Showcasing the phenolic content values for different plant species

Plant Species	Phenolic content (mg_GAE_/g)	References
*Green Tea Leaves*	38.70	Ateş et al. (2022)
*Passiflora Edulis Sims seeds Fruits*	9.25	Samudin et al. (2022)
*Apium Graveolens Leaves*	0.1921	Salamatullah et al. (2021)
*Sonneratia ovata Back*	0.104	Astuti et al. (2023)
*Carica papaya leaves*	21.58	Yap et al. (2020)

### Characterization of P-NZVI nanoparticles

#### UV–vis spectral analysis

**Fig. 3:**
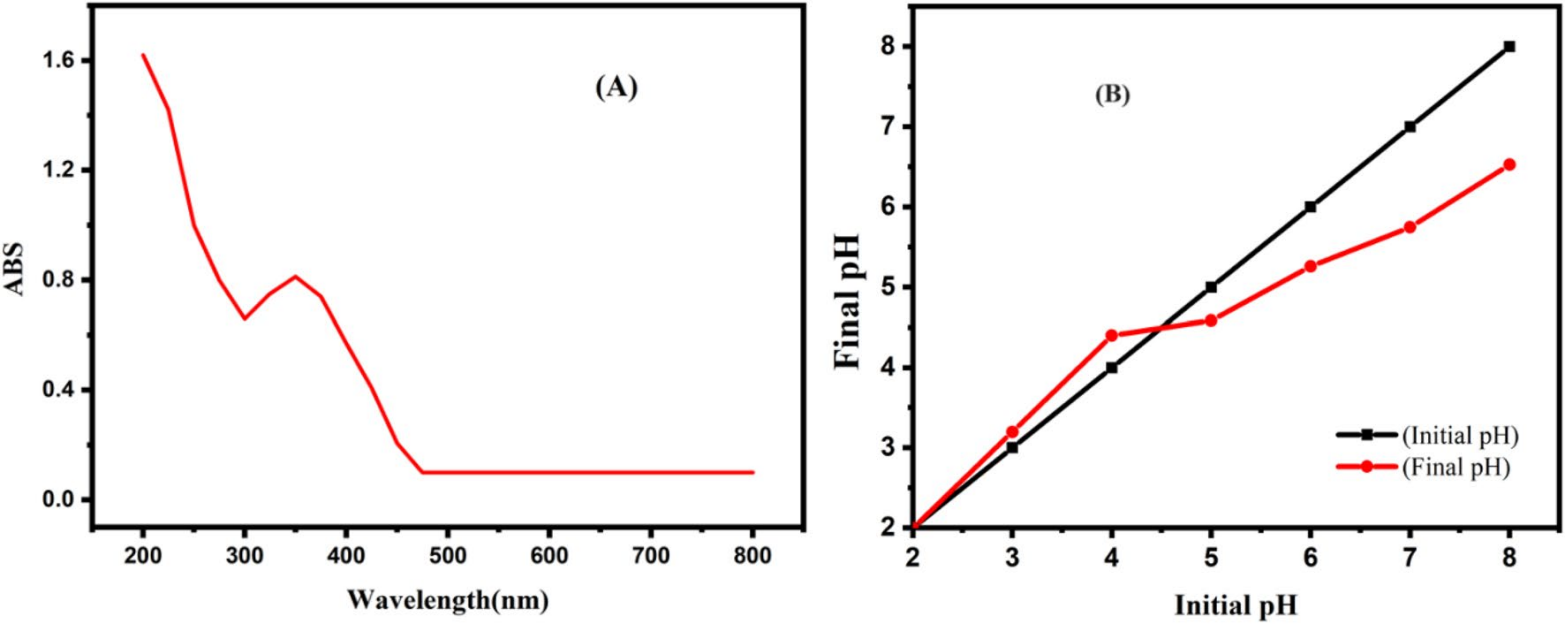
**A** UV-visible analysis and **B** pH_ZPC_ of P-NZVI

As previously mentioned, the solution’s color changed during nanoparticle formation. The observed color change in the mixture of extracts after their addition to the iron solution indicates the formation of nanoparticles (Abdelfatah et al. 2021). Fig. 3A presents the synthesized nanoparticles’ absorption spectra, with an intense peak at 350 nm confirming the production of P-NZVI. The zero-valent iron state was assigned a peak range of 200–450 nm, which is entirely consistent with the outcomes of other research utilizing different plants, including seeds, peels, and leaves extracts (Desalegn et al. 2019; Naveed et al. 2023).

#### ***pH***_***ZPC***_ analysis

In this experiment, 50 mL of a 0.1 M NaCl solution was distributed into seven different beakers. The pH of each solution was adjusted to 2, 3, 4, 5, 6, 7, and 8. Subsequently, 0.05 g of iron nanoparticles was added to each beaker, and the mixtures were left undisturbed for 48 h. A parallel control experiment was conducted without adding iron nanoparticles. After 48 h, the pH of the solutions was measured, and the change in pH was plotted on a graph—the point where the final pH of P-NZVI intersected with the control was identified as the pH_ZPC_ (zero-point charge) of the nanoparticles. The pH_ZPC_ of P-NZVI was determined to be 4.8 (Fig. 3B). This finding indicates that the surface charge of P-NZVI is neutral at pH 4.8 but becomes positive below this pH value. Conversely, when the solution pH exceeds the pH_ZPC_, the surface of P-NZVI will carry negative charges.

#### DLS and Zeta potential analysis

At 25 °C, the values of dynamic light scattering (DLS), Zeta potential (ZP), and polydispersity index (PDI) were measured. The mean hydrodynamic diameter of the nanoparticles is 72 nm. Assemblages of polyphenols surrounding the nanoparticles, on the other hand, may be responsible for the relatively high values of particle sizes (Karavasilis and Tsakiroglou 2019). ζP is a key parameter for evaluating the stability of nanoparticles in aqueous solutions. Negative Zeta potential values confirm a high degree of stability in the system (Vilardi et al. 2019). The ζP value of P-NZVI is −27.2 mV, confirming the stability of P-NZVI. The PDI value ranging from 0 to 1, and indicates the width or extent of the particle size distribution. Values less than 0.1 are considered for monodisperse particles, whereas values higher than 0.1 are considered for polydispersity distributions (Ocampo Silva). The PDI of P-NZVI is 1, suggesting a polydispersity particulate system.

#### Morphology and size of P-NZVI

**Fig. 4:**
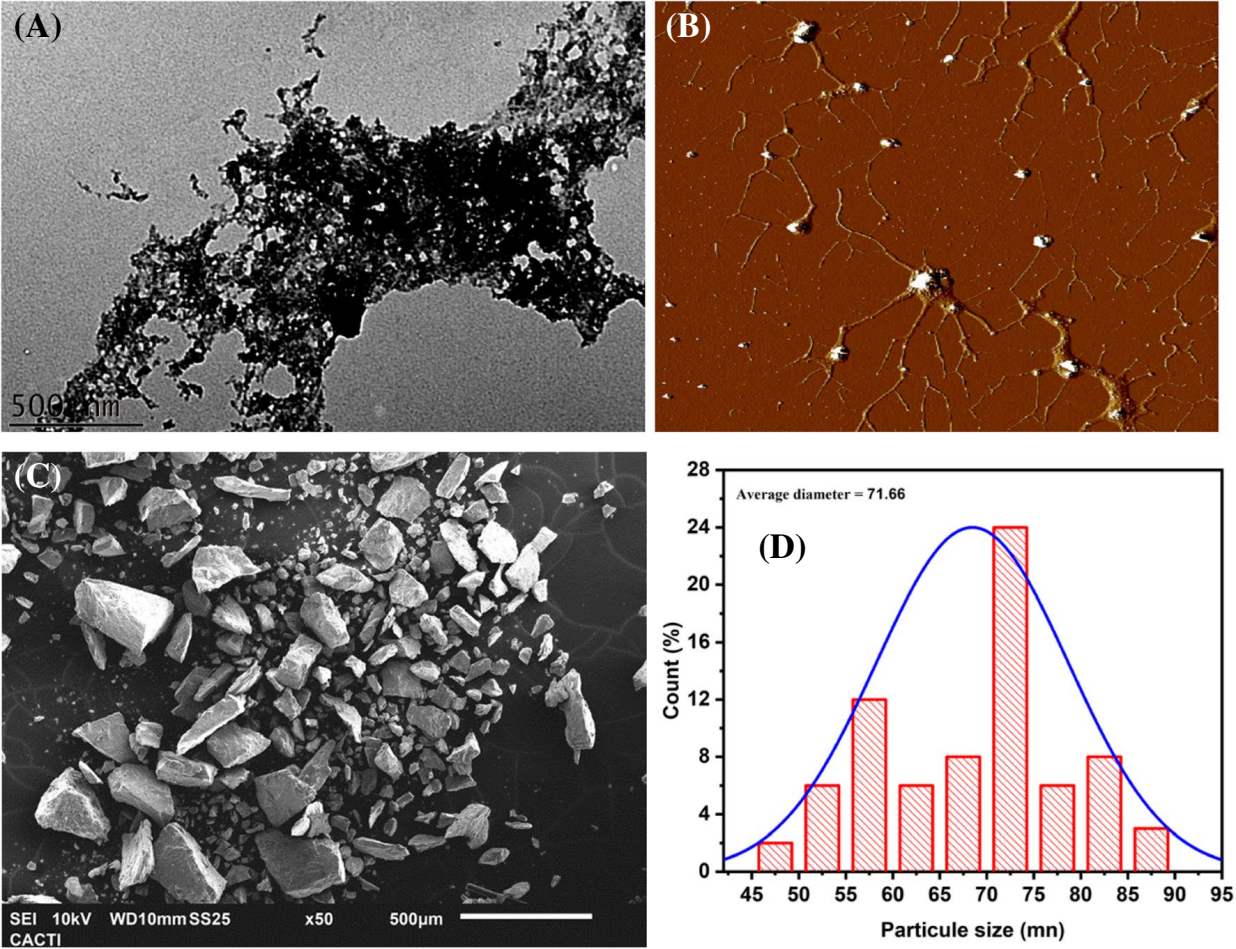
TEM image (**A**), AFM image (**B**), SEM image (**C**), and (**D**) diagram of the P-NZVI size distribution

Transmission electron microscopy (TEM), scanning electron microscopy (SEM), and atomic force microscopy (AFM) were conducted to elucidate the particle size, shape, and topology of the synthesized P-NZVI. The TEM images (Fig. 4A, D) reveal that P-NZVI tends to form chain-like aggregates with an average particle size of 70–75 nm. Besides its spherical shape, P-NZVI exhibits other irregular shapes. The AFM technique provides a detailed image of the atomic surface of the chain-like aggregates (Fig. 4B), corroborating the findings from the TEM images. The nanoscale particle size contributes to the chain’s high specific surface area and reactivity with contaminants (Bagbi et al. 2017). Furthermore, as evident from the TEM images, the surface of the synthesized P-NZVI is enveloped by a transparent layer. This layer acts as a capping and stabilizing agent, preventing P-NZVI from agglomerating. It plays a crucial role in enhancing the dispersion and stability of P-NZVI, serving as a fundamental component of green-synthesized NZVI (Hamad and El-Sesy 2023; Panić et al. 2023). In the present experiment, P-NZVI was synthesized, and as depicted in Fig. 4C, SEM images indicate a predominantly granular structure with a spherical form. These SEM images provide additional evidence which supports the observations obtained from TEM. However, an analysis of the size distribution and mean size of P-NZVI based on TEM photomicrographs revealed that the particle size distribution ranged between 45 and 90 nm, with the most frequent particle size being 71.66 nm.

#### FTIR analysis

**Fig. 5:**
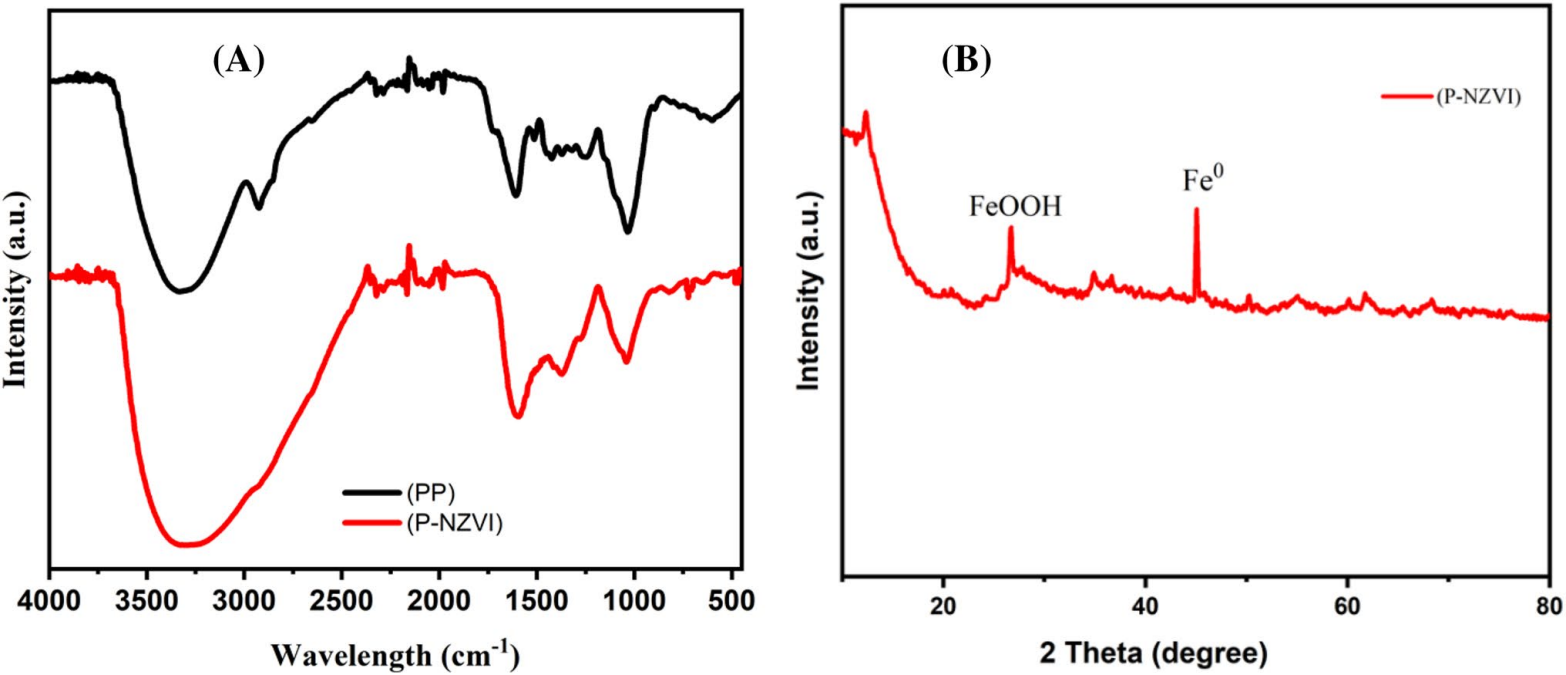
**A** FTIR spectra of P-NZVI and PP raw materials, **B** XRD of P-NZVI

FTIR spectra of P-NZVI and PP raw material are shown in Fig. 5A. The peak of PP displays broadband stretching vibrations at 3363 cm^−1^ for O–H, indicating the presence of polyphenols (Abdel-Aziz et al. 2019). The presence of phenolic compounds in raw plants is significant because these compounds are frequently attributed to the direct reduction of iron(III) to iron(0) and the formation of a cladding layer on the surface of NZVI (Samadi et al. 2021). The peak intensity at 3300 cm^−1^ was slightly reduced for the P-NZVI after the reduction, indicating the involvement of polyphenolic substances in the PP extract in the production of P-NZVI. The absorption peak at 1534 cm^−1^ is associated with the aromatic ring stretching vibration in the phenolic compound, and the peak located at 1052 cm^−1^ is assigned to C–O, C–O–H, and symmetric and asymmetric C–O–C groups present in P-NZVI (Li et al. 2022b; Ma et al. 2022) and PP (Abderrahim et al. 2023). Furthermore, in P-NZVI, the weak absorption bands at 795 and 528 *cm*^−1^ are attributed to the Fe–O stretching vibration of Fe oxide (Li et al. 2019; Hoa et al. 2020). Moreover, the primary characteristic peaks in the FTIR spectrum of P-NZVI closely resemble those of the PP, indicating that the surface of P-NZVI is coated with the active components of the PP.

#### XRD analysis

The XRD analysis of P-NZVI (Fig. 5B) unveiled a characteristic peak at approximately 45^°^, corresponding to the (110) plane. This peak has been identified as the characteristic peak of zero-valent iron (Koliana 2022; Le et al. 2022). Iron hydroxides are responsible for the characteristic peak at 2θ = 31.82^°^ corresponding to the (111) plane (Huang et al. 2014; Ebrahiminezhad et al. 2017). The peak observed in the XRD pattern was associated with the iron oxides formed during the oxidation of iron with oxygen. Additionally, the peaks at 2θ = 13.49^°^ and 14.52^°^ are attributed to organic matter present on the surface of P-NZVI (Soliemanzadeh et al. 2016).

#### BET analysis

**Fig. 6:**
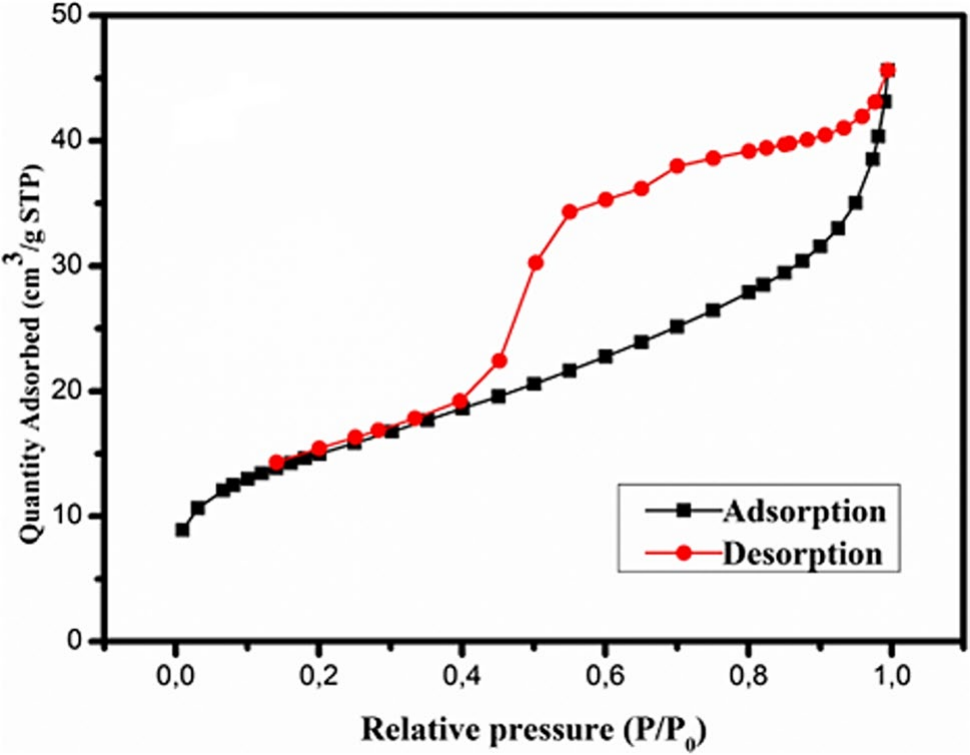
N_2_ adsorption-desorption isotherm of the P-NZVI

BET analysis enables the precise assessment of the specific surface area by measuring the material area through nitrogen multilayer adsorption as a function of relative pressure using a fully automated analyzer (Ren et al. 2023). Surface measurement was conducted at 77 K using high-purity nitrogen. The nitrogen adsorption-desorption isotherm plot of P-NZVI is depicted in Fig. 6. According to the International Union of Pure and Applied Chemistry (IUPAC), a mesoporous material contains pores with widths ranging from 2 to 50 nm. Additionally, microporous pores have dimensions less than 2 nm, while macroporous pores have dimensions greater than 50 nm (Tella et al. 2022). The nitrogen adsorption-desorption isotherm plot of P-NZVI corresponds to a typical type IV isotherm, indicating the presence of a mesoporous structure according to the IUPAC classification (Du et al. 2019; Li et al. 2022a). The BET surface area, average pore width, and total pore volume of P-NZVI were identified to be 52.69 m^2^/g, 4.68 nm, and 0.06 cm^3^/g, respectively.

### Adsorption studies

#### Effect of pH

**Fig. 7:**
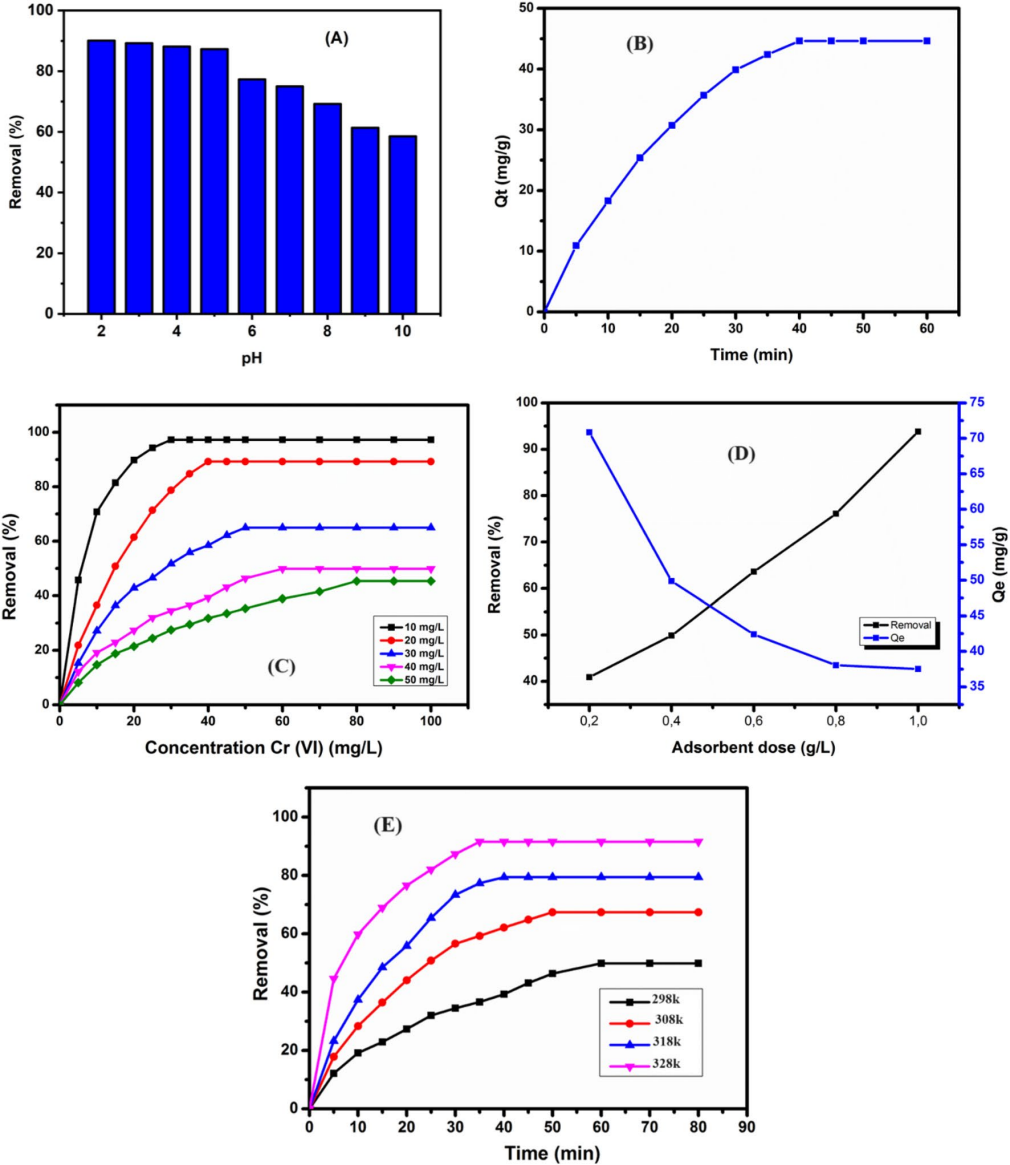
**A** Effect of pH of Cr(VI) adsorption, **B** effect of contact time of Cr(VI) adsorption, **C** effect of initial concentration of Cr(VI) adsorption, **D** effect of dose adsorbent of Cr(VI) adsorption, **E** effect of temperature of Cr(VI) adsorption

In this study, we examined Cr(VI) adsorption efficiency at different pH values ranging from 2 to 10 using HCl (0.1N) or NaOH (0.1N). Twenty milligrams of the adsorbent was stirred in 50 mL of a 20 mg/L Cr(VI) solution for 60 min at room temperature. The findings presented in Fig. 7A reveal that the Cr(VI) adsorption capacities exhibit a relatively consistent trend from pH 2 to 5, with values clustering closely together. However, beyond pH 5, there is a noticeable decline in adsorption capacity. Notably, at pH 5, the maximum removal efficiency reaches 89.3%, corresponding to adsorption capacity of 44.65 mg/g.

Since the ionization of the surface of P-NZVI adsorbent when the pH shifts from lower to higher values, the adsorption capacity changes due to the electrostatic interactions between the adsorbates and adsorbents (WooáLee and BináKim 2011; Zou et al. 2016). This phenomenon can be attributed to increased iron corrosion at lower pH values, favoring the hydrogenation reaction, and the formation of a passive film of iron hydroxide at higher pH values, promoting the fixation of pollutant species on the surface of the adsorbent. The *pH*_*ZPC*_ for P-NZVI was measured at 4.8 (Fig. 3B), indicating a positively charged surface below this pH. Hence, at the initial pH of 5, electrostatic attraction facilitated Cr(VI) adsorption onto the P-NZVI surface (Mortazavian et al. 2018; Zhou et al. 2020). The impact of pH on Cr(VI) removal by P-ZVI could be associated with the reduction of Cr(VI) to Cr(III) at low pH (Eqs. 9 to 11) (Djellabi et al. 2017), while at higher pH, precipitation of Fe(OH)_2_/Fe(OH)_3_ blocks reactive sites, decreasing process efficiency (Xu et al. 2021). These findings emphasize the importance of maintaining a pH value of 5 for effective Cr(VI) adsorption by P-NZVI.

9 $$2{\mathrm{H}\mathrm{CrO}}_4^{-}+3{\mathrm{Fe}}^0+14\ {\mathrm{H}}^{+}\to 3{\mathrm{Cr}}^{3+}+2{\mathrm{Fe}}^{3+}+8{\mathrm{H}}_2\mathrm{O}$$

10 $${\mathrm{H}\mathrm{CrO}}_4^{-}+3{\mathrm{Fe}}^{2+}+3\ {\mathrm{H}}^{+}\to {\mathrm{Cr}}^{3+}+3{\mathrm{Fe}}^{3+}+2{\mathrm{H}}_2\mathrm{O}$$

11 $${\mathrm{Cr}\mathrm{O}}_4^{2-}+{\mathrm{Fe}}^0+8\ {\mathrm{H}}^{+}\to {\mathrm{Cr}}^{3+}+3{\mathrm{Fe}}^{2+}+4{\mathrm{H}}_2\mathrm{O}$$

#### Effect of contact time

The influence of reaction time on Cr(VI) adsorption by P-NZVI is depicted in Fig. 7B under pH 5 conditions, a mass of adsorbent of 0.4 g/L, and an initial concentration of 20 mg/L at 298 K. Initially, the adsorption of Cr(VI) exhibited a rapid increase, reaching a peak at 40 min. However, there was no significant rise in adsorption afterward, suggesting an adsorption equilibrium at 40 min. The maximum efficiency for removing Cr(VI) was 89.3% at 40 min, with a corresponding maximum adsorption capacity of 44.47 mg of Cr(VI)/g of P-NZVI. The relatively quick adsorption equilibrium, due to fast Cr(VI) fixation, could be a technological and economic advantage at a large scale.

#### Effect of initial concentration of Cr(VI)

Fig. 7C illustrates the effect of the initial concentration of Cr(VI) on adsorption under pH 5 conditions, with an adsorbent dose = 0.4 g/L, for 90 min at *T* = 298 K. With an increase in the initial concentration of Cr(VI) from 10 to 50 mg/L, the removal efficiency of Cr(VI) by P-NZVI decreased from 97.3 to 45.4%, respectively. Conversely, the maximum adsorption capacity increased from 24.32 to 56.75 mg of Cr(VI) per gram of P-NZVI. The rapid removal of Cr(VI) during the initial stage of the reaction With P-NZVI suggests that the large specific area of nanoparticles promotes the reaction between Cr(VI) and P-NZVI. At a low dose, P-NZVI exhibits high removal efficiency. However, as the concentration of Cr(VI) increases, a gradient forms between the sorbent and the chrome ions, serving as a potential driving force for ion exchange. The decrease in the removal percentage of Cr(VI) with increasing concentration from 10 to 50 mg/L is attributed to the rapid exhaustion of adsorption sites and a charge balance between the active functional groups and Cr(VI) ions (Kuppusamy et al. 2016). The adsorption capacity reaches a maximum of 20 mg/L of Cr(VI). Beyond this concentration, the amount of adsorbed Cr(VI) remains constant, indicating the maximum adsorption capacity of P-NZVI towards Cr(VI) removal.

#### Effect of adsorbent dosage

The removal of Cr(VI) was investigated by varying the adsorbent dosage from 0.2 to 1 g/L at an initial Cr(VI) concentration of 40 mg/L, pH 5, for 90 min (Fig. 7D). The maximum adsorption of Cr (VI) by P-NZVI was observed at a dose of 1 g/L, reaching 93.8%. With an additional dosage (1 g/L) of P-NZVI, rapid adsorption and equilibrium were achieved within 40 min, while a lower dosage (0.2 g/L) required 80 min to reach equilibrium. As the nanoparticle dose increased, the adsorption capacity decreased, likely due to nanoparticle aggregation, reducing the accessible surface area for adsorption (Daneshvar and Hosseini 2018).

#### Effect of temperature

The impact of temperature on Cr(VI) adsorption was investigated under optimal conditions. The results are depicted in Fig. 7E. At a P-NZVI dosage of 0.4 g/L, an initial Cr(VI) concentration of 40 mg/L, and a pH of 5, the efficiency of Cr(VI) removal increased with rising temperature, ranging from 49.87% at 298 K to 91.6% after 60 min at 328 K. The enhanced adsorption-reduction capability at higher temperatures can be attributed to increased energy for the reaction, promoting molecular mobility and accelerating the diffusion and transfer of Cr(VI) into the reactive sites of P-NZVI (Zheng and Duan 2022). This makes Cr(VI) more easily absorbed and reduced by P-NZVI. As a result, P-NZVI achieved greater Cr(VI) removal effectiveness at higher temperatures.

### Kinetic and isothermal studies

#### Adsorption kinetic

**Fig. 8:**
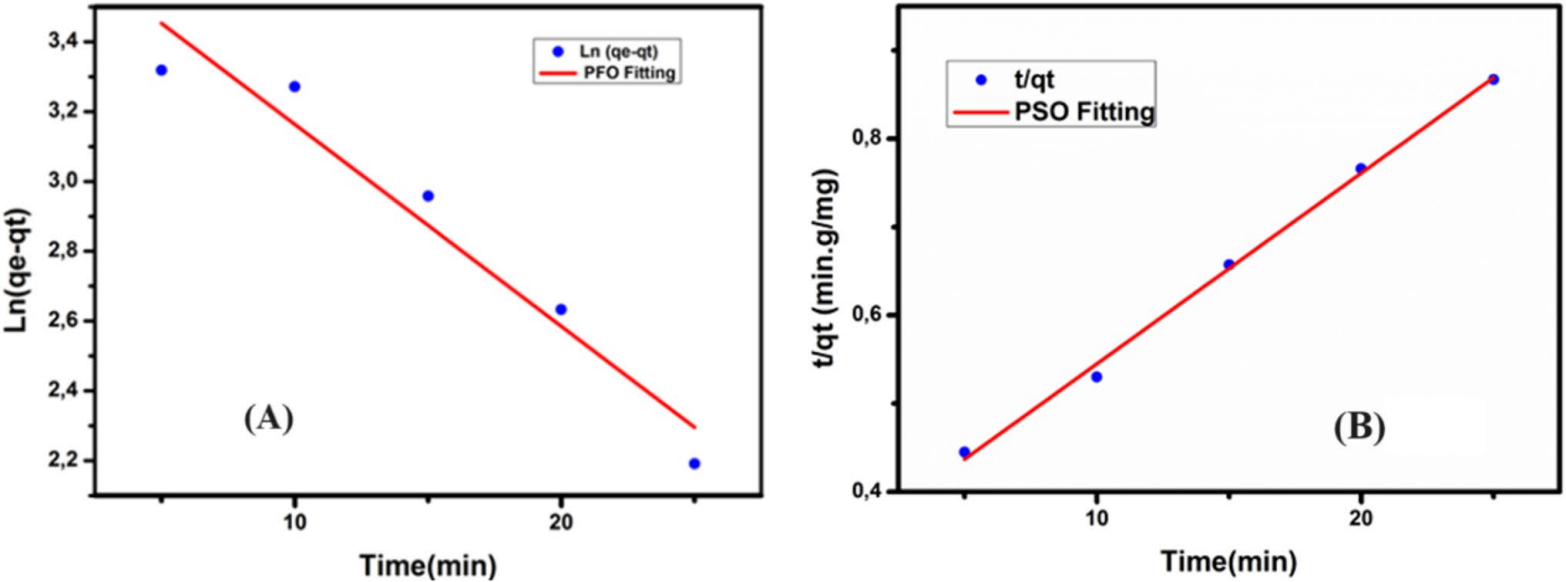
**A** PFO and **B** PSO kinetic models (pH 5, [Cr(VI)] = 20mg/L, dose adsorbent = 0.4 g/L, *V*=50 mL, *T* = 25 °C)

Kinetic experiments provide insights into the mechanisms governing an adsorption process (Islam et al. 2020). Various kinetic adsorption models were employed to analyze the kinetic data, including PFO, PSO, and IPD models. The equations for the linear kinetic models and their parameter definitions are presented in Table 3. The estimation of PFO parameters involves plotting Ln (*q*_*e*_ − *q*_*t*_) against time (Fig. 8A), while the analysis of PSO parameters involves plotting $\frac{t}{q_t}$ against time (Fig. 8B). However, the IPD model’s plot did not pass through the origin, displaying a multi-linear curve. This suggests that IPD is just one phase in the adsorption process and cannot be considered the sole rate-limiting step.

**Table 3:** Kinetics parameters for PFO and PSO

Models		Pseudo-first-order PFO			Pseudo-second-order PSO		
Equations		Ln(*q*_e_ − *q*_*t*_) = Ln *q*_e_ − *K*_1_*t*			$\frac{t}{q_t}=\frac{1}{k_{2\mathrm{p}}{q}_{\mathrm{e}}^2}+\frac{t}{q_{\mathrm{e}}}$		
*C*_0_ (mg/l)	*q*_e, exp_ (mg/g)	*q*_e, cal_ (mg/g)	*k*_1_ (min^−1^)	*R*^2^	*q*_e, cal_ (mg/g)	*k*_2_(g/mg·min)	*R*^2^
20	44.47	40.44	0.0454	0.971	45.28	0.02	0.996

The kinetic parameters were determined by plotting the kinetic models under optimal pH conditions (pH 5) and a nanoparticle dosage of 4 g/L at room temperature. The regression coefficients (*R*^2^) validated the correlation between the predicted kinetic model values and the experimental results. The PSO kinetic model was found to best characterize the kinetic experimental data, as indicated by *R*^2^ values approaching unity. This suggests that Cr(VI) adsorption on P-NZVI follows a chemical adsorption route, where the adsorption process is controlled by chemisorption involving electron exchange between the adsorbate and the adsorbent (Tapouk et al. 2020; Gao et al. 2022)**.**

#### Adsorption isotherm

**Fig. 9:**
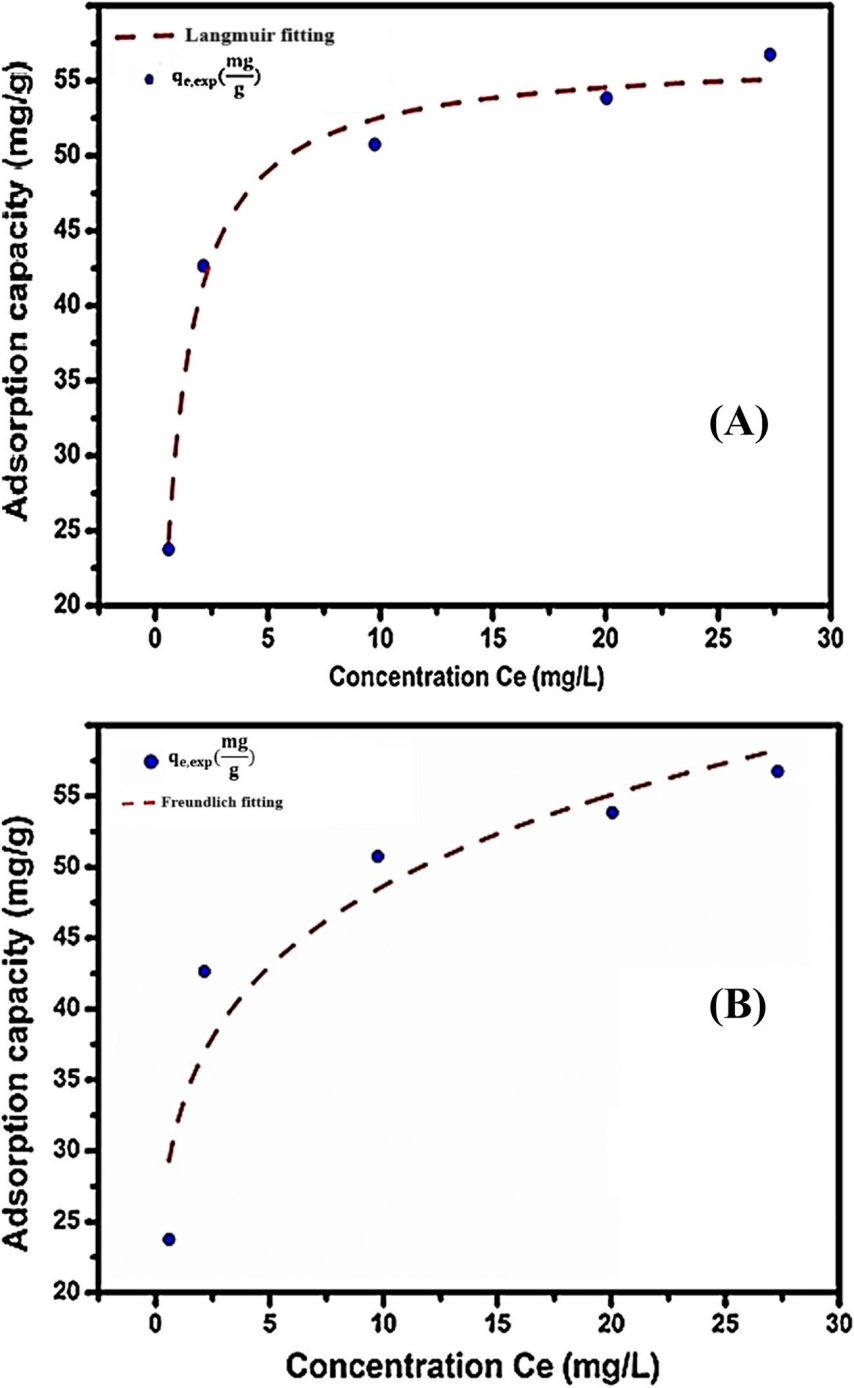
**A** Langmuir isotherm plot and **B** Freundlich isotherm plot (pH 5, [Cr(VI)] = 20mg/L, dose adsorbent = 0.4 g/L, *V*=50 mL, *T* = 25 °C)

The Langmuir and Freundlich models’ isotherm parameters and *R*^2^ values are obtained from the slopes and intercepts through the plotting of ${q}_{\mathrm{e}}={K}_{\mathrm{F}}{C}_{\mathrm{e}}^n$ (Fig. 9A) and $\frac{C_{\mathrm{e}}}{q_{\mathrm{e}}}=\frac{C_{\mathrm{e}}}{q_{\mathrm{m}}}+\frac{1}{q_{\mathrm{m}{\mathrm{k}}_{\mathrm{L}}}}$ (Fig. 9B), respectively. Fig. 9 displays the isotherm plots for furfural adsorption by P-NZVI at a pH of 5, 0.4 g/L dose, and room temperature.

Table 4 presents the results obtained from the Langmuir and Freundlich isotherm models for furfural adsorption by P-NZVI. The Langmuir isotherm model yielded an *R*_L_value of 0.056, indicating favorable adsorption, while the Freundlich isotherm model resulted in an *n* value of 5.591, suggesting unfavorable adsorption. The Langmuir model assumes an energetically homogeneous surface with uniformly distributed adsorption sites (Zhang et al. 2014; Ayuba et al. 2019).

**Table 4:** Equilibrium parameters for Langmuir and Freundlich

Langmuir				Freundlich		
$\frac{C_{\mathrm{e}}}{q_{\mathrm{e}}}=\frac{C_{\mathrm{e}}}{q_{\mathrm{m}}}+\frac{1}{q_{\mathrm{m}{\mathrm{k}}_{\mathrm{L}}}}$				${q}_{\mathrm{e}}={K}_{\mathrm{F}}{C}_{\mathrm{e}}^n$		
*q*_m_(mg/g)	*K*_L_(L/mg)	*R*^2^	*R*_L_	*k*_F_(L/g)	*n*	*R*^2^
56.68	1.272	0.9851	0.056	32.24	5.591	0.8620

### Adsorption thermodynamics

The thermodynamic analysis sheds light on the adsorption process of Cr(VI) on P-NZVI. Table 5 shows the decrease in ∆*G*^°^ with increasing temperature indicates that adsorption becomes more favorable at higher temperatures, consistent with similar observations in studies by S. Rawat et al. (Rawat and Singh 2021). The positive ∆*H*^°^ value suggests an endothermic process, with a high value indicating that chemisorption is the primary rate-controlling mechanism (63.41 kJ/mol) (Mahmoudian et al. 2023). Chemisorption involves strong chemical bond formation between P-NZVI and Cr(VI) (Sumalinog et al. 2018)—additionally, the positive ∆*S*^°^ value ( 0.277 kJ/ mol. K) indicates an increase in the degree of freedom at the P-NZVI interface during adsorption (Thabede et al. 2020), implying higher affinity and increased randomness at the solid surface contact (Liu et al. 2019; Maamoun et al. 2021).

**Table 5:** Thermodynamic parameters at different temperatures

Temperature (K)	lnK_D_	∆*G*^°^ (KJ/mol)
298.15	7.997	−20.321
308.15	8.531	−21.930
318.15	9.175	−24.276
328.15	10.214	−27.863

## Recycle of adsorbent P-NZVI on Cr(VI) adsorption

**Fig. 10:**
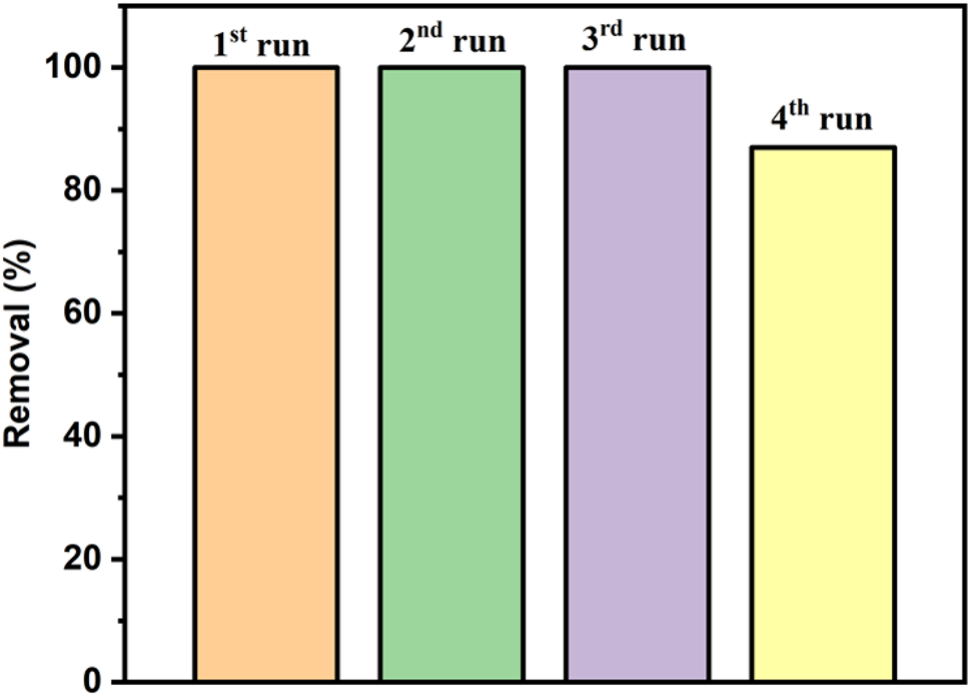
Recycle of P-NZVI adsorbent

Fig. 10 demonstrates the successful reuse of P-NZVI adsorbent for four consecutive adsorption cycles, with an effectiveness of over 87%. This promising outcome underscores the potential for regenerating and recycling the adsorbent, contributing to the cost-effectiveness and sustainability of the Cr(VI) removal process using P-NZVI. Effective regeneration and recycling of adsorbents are critical factors in practical applications, particularly in large-scale or continuous treatment systems.

## Comparison of the maximum adsorption capacity (***q***_***m***_) of NZVI to Cr(VI) adsorption

The statement highlights that the comparison of the maximum adsorption capacity (*q*_*m*_) of P-NZVI with other adsorbents for Cr(VI) adsorption, presented in Table 6, demonstrates the superior effectiveness of the P-NZVI composite. In addition to the sorption performance of P-NZVI, the sustainable and low-cost synthesis of the material using locally available wastes could make P-NZVI a strong material candidate for water treatment at large scale.

**Table 6:** Comparisons of the maximum adsorption capacity (*q*_*m*_) of NZVI for Cr(VI)

Adsorbent	Raw plants	*q*_m_ (mg/g)	References
ZVIN	*Catharanthus roseus flower*	9.09	Sravanthi et al. (2019)
FeNPs	*Yali pear peels*	43.99	Rong et al. (2022)
nZVI@Fe3O4@HMIMPF6	*Camellia sinensis Leaves*	56.49	Mousazadeh et al. (2021)
CL-FeNPs	*Citrus limetta peels*	33	Dalal and Reddy (2019)
nZVI	*Korla fragrant pear peel*	46.62	Rong et al. (2020)
P-NZVI	*Palm petiole*	44.47	This study

Even though the synthesis process of P-NZVI is a green, low-cost route, and the material shows high efficiency in removing Cr (VI) from water, P-NZVI should be further investigated to understand its performance at large scale, and to know its long-term chemical stability in the presence of different co-existing chemical and biological species in real wastewaters.

## Conclusion

The study finding proves the effectiveness of as-synthesized P-NZVI derived from PP extract as a sustainable adsorbent for removing Cr(VI) from water. P-NZVI’s characteristics were defined by different physicochemical techniques. The performance of P-NZVI was estimated under various factors. The adsorption capacity was found to be 44.47 mg/g within 40 min under these conditions: initial concentration of 40 mg/L, pH of 5, and P-NZVI dosage of 1 g/L. The adsorption mechanism was in accordance with PSO kinetic and Langmuir models, suggesting a combination of chemisorption and monolayer adsorption processes. The process was demonstrated to be both spontaneous and endothermic by thermodynamic analysis. The reusability study shows that P-NZVI can be regenerated and used for four cycles without notable reduction in the adsorption effectiveness.

These results highlight the feasibility of using Palm Petiole extract as a green-reducing agent to prepare P-NZVI with a high adsorption ability to remove Cr(VI) from water. This approach supports the concept of agro-waste valorization, which fits with the context of the circular economy paradigm and environmental sustainability. In the next studies, the performance of P-NZVI and long-term stability in actual wastewater conditions will be investigated to remove Cr(VI) or other types of pollutants. The application of P-NZVI as a Fenton heterogeneous catalyst will be studied in the oxidation of organic pollutants. On top of that, the P-NZVI adsorption process and other technologies could be addressed to promote the purification of actual wastewater.
